# Quasi-Drugs Developed in Japan for the Prevention or Treatment of Hyperpigmentary Disorders. *Int. J. Mol. Sci.* 2010, *11*, 2566–2575

**DOI:** 10.3390/ijms11072699

**Published:** 2010-07-12

**Authors:** Hideya Ando, Mary S. Matsui, Masamitsu Ichihashi

**Affiliations:** 1 Skin Aging and Photo-aging Research Center, Doshisha University, Kizugawa, Kyoto 619-0225, Japan; E-Mail: mm_ichihashi@hotmail.com; 2 Kobe Skin Research Institute, Kobe, Hyogo 650-0047, Japan; 3 Biological Research Division, The Estee Lauder Companies Inc., Melville, New York 11747, NY, USA; E-Mail: mmatsui@Estee.com

One additional skin lightening or whitening quasi-drug (QD) has been developed and officially approved by the Ministry of Health, Labor and Welfare of Japan. The active ingredient niacinamide should be included in this review [1]. Its mechanism of skin lightening is based on the inhibition of melanosome transfer from melanocytes to keratinocytes. Niacinamide is listed in Table 1, which classifies compounds according to the mechanism of skin lightening QDs registered in Japan.

## 2.15. Niacinamide (Obtained by Procter & Gamble Company in 2007)

Niacinamide (also termed nicotinamide), a derivative of vitamin B_3_, has been shown to act as an anti-inflammatory agent in acne [2]. Niacinamide had no effect on the tyrosinase activity and melanin synthesis of cultured normal human melanocytes, however, it was found that niacinamide significantly decreased hyperpigmentation, such as melasma and solar lentigines, via inhibition of melanosome transfer from melanocytes to keratinocytes [3,4].

## Figures and Tables

**Table 1.** Mechanistic classification of skin lightening QDs approved by the MHLW of Japan.

***Target***	***Mechanism***		***Detail***	***Skin Lightening QD***	

**Melanocyte**	Inhibition of tyrosinase activity		Anti-oxidation	Ascorbic acid/derivatives	

			Chelating copper atoms	Kojic acid	Ellagic acid

			Competitive inhibition	Arbutin 4MSK	Rucinol® 4-HPB
	
		Decrease of tyrosinase protein level	Acceleration of Tyr degradation	Linoleic acid	

			Inhibition of Tyr maturation	Magnolignan®	

**Keratinocyte**	Inhibition of KC-MC signaling		Inhibition of UV inflammation	Chamomilla extract Tranexamic acid/derivative	

**Melanocyte and Keratinocyte**	Inhibition of melanosome transfer		Inhibition of melanin dispersion	Niacinamide	

**Epidermis**	Acceleration of epidermal turnover		Desquamation of melanin	Placental extract Adenosine mono-phosphate	

KC: keratinocyte; MC: melanocyte; Tyr: tyrosinase; UV: ultraviolet light.

## References

[1] Ando H Matsui MS Ichihashi M Quasi-drugs developed in Japan for the prevention or treatment of hyperpigmentary disorders Int. J. Mol. Sci 2010 11 2566 2575 20640168 10.3390/ijms11062566 PMC2904932

[2] Shalita AR Smith JG Parish LH Sofman MS Chalker DK Topical nicotinamide compared with clindamycin gel in the treatment of inflammatory acne vulgaris Int. J. Dermatol 1995 34 434 437 7657446 10.1111/j.1365-4362.1995.tb04449.x

[3] Hakozaki T Minwalla L Zhuang J Chhoa M Matsubara A Miyamoto K Greatens A Hillebrand GG Bissett DL Boissy RE The effect of niacinamide on reducing cutaneous pigmentation and suppression of melanosome transfer Br. J. Dermatol 2002 147 20 31 12100180 10.1046/j.1365-2133.2002.04834.x

[4] Greatens A Hakozaki T Koshoffer A Epstein H Schwemberger S Babcock G Bissett D Takiwaki H Arase S Wickett RR Boissy RE Effective inhibition of melanosome transfer to keratinocytes by lectins and niacinamide is reversible Exp. Dermatol 2005 14 498 508 15946237 10.1111/j.0906-6705.2005.00309.x

